# Perceptions of medical students towards the role of histology and embryology during curricular review

**DOI:** 10.1186/s12909-023-04019-4

**Published:** 2023-01-30

**Authors:** Bruno Daniel Carneiro, Daniel Humberto Pozza, Isaura Tavares

**Affiliations:** Unit of Experimental Biology, Department of Biomedicine, Faculty of Medicine, University of Porto, Porto, Portugal

**Keywords:** Medicine, Students, Histology, Embryology, Biopathology, Teaching, Virtual microscopes

## Abstract

**Background:**

The continuous changes in the medical education to prepare medical doctors for the future requires updates in medical curriculum. However, the perspectives of the medical students are not frequently considered during the revision of the medical curriculum. In parallel with the process of defining and adjusting the medical curriculum, a large survey was performed to inquire the perspectives of the medical students at the Faculty of Medicine of the University of Porto (FMUP), Portugal, about the role of Histology and of Embryology.

**Methods:**

Medical students at FMUP (Portugal) completed a structured and anonymous online questionnaire about the subjects Histology and Embryology. The questionnaire was prepared using questions of previous surveys performed in Europe, including another Portuguese medical school, and additional questions that were specifically prepared to this study. The questions referred to teaching methods, clinical relevance, use of virtual (digital) microscopes and association of Histology and Embryology with other subjects of the medical curriculum.

**Results:**

Four hundred and sixty-two students participated in the study. The students in clinical years were more likely to recognise the clinical relevance of Histology (*p* = 0.016) and Embryology (*p* < 0.001). Students agree that teaching of these subjects would benefit from a clinical orientation (89% for Histology; 90% for Embryology). Students highlighted that Histology is crucial to understand Biopathology and agree (75%) that an integration of Histology with Biopathology could be considered in the medical curriculum. Most students (55%) agree that slide microscopes are more useful than virtual microscopes.

**Conclusions:**

Our study contributes to the debate about the evolution of medical curriculum. Gathering the medical students’ perceptions using large surveys such as that performed in the present study may be useful to adapt the methods of teaching which may increase the motivation of the students. In the case of Histology and Embryology at the FMUP (Portugal) providing more clinically oriented teaching may be useful to motivate the students. Students of clinical years have strong clinical perspectives of Histology and Embryology and their enrolment in teaching of Histology and Embryology can also contribute to increase motivation of younger students. Consulting and involving medical students in the development of the medical curriculum can be positive and students should be more responsible and engaged in building their own education.

**Supplementary Information:**

The online version contains supplementary material available at 10.1186/s12909-023-04019-4.

## Background

The development of medical sciences has been encouraging the emergence of new philosophies and approaches to medical education [1]. Student-centered philosophies in which the student engagement in learning increased, pursuing personal interests by increasing the importance of elective courses and an anticipation of clinically oriented contents are amongst the used methods. Increasing the use of digital resources, simulation training, encouraging teamwork early on medical training and fostering the development of soft skills are also increasing in medical courses. Over the past decades there have been major reforms in undergraduate medical education [2]. The explosion of scientific knowledge that led to the introduction of new disciplines into medical curriculum is one of the reasons for the reforms [3]. However, it is important to decide if teaching more classical subjects, such as Histology and Embryology, should be also deeply changed, namely by merging these subjects with others and creating new disciplines. Furthermore, medical educators realised that factual knowledge in the first years of the medical curriculum is frequently not sufficient to produce competent physicians and that basic medical training should focus on the clinical relevance [4]. Finally, the first years of the medical courses are important in setting the motivation of the students for a long educational process [5].

Most medical schools are moving towards a teaching methodology that better prepares students for clinical practice [6, 7], with the implementation of a more clinically-oriented teaching of basic sciences. The demand for innovations in medical education along with development of alternative assessment methods has popularised the idea of the integrated curriculum [8]. In fact, since 1990, medical education in basic medical sciences disciplines have moved to interdisciplinary integrated curriculum [9].

Histology and Embryology are two classic medical subjects that in Latin countries have the tradition of being associated. Embryology remains an important subject to understand normal development, as well as micro and macroanatomy, helping to better understand clinical abnormalities. However, as a subject, Embryology it is not easy to teach and can be easily overlooked by medical students [10]. Histology is also an important tool in medicine, bridging the gap between the normal microstructure of organs and tissues and their pathological changes [11].

In Portugal, there are nine medical schools, but the medical education system is diverse. In our medical school, the Faculty of Medicine of the University of Porto (FMUP), the first 3 years of the course focuses on the transmission of knowledge associated with the basic sciences, which include Histology and Embryology, but also subjects such as Anatomy, Physiology, Biopathology, Microbiology or Immunology. In the last 3 years of the course the students are deeply engaged on the progressive acquisition of clinical skills, in the different medical specialities. Regarding Histology and Embryology, different teaching methods have been used by the medical schools. The classical teaching of Histology and Embryology is changing and several medical schools in different countries have analysed how teaching of Histology and of Embryology recently evolved [12 – 15]. For example, Histology and Embryology are taught as stand-alone course in most cases and some universities are trying to modernise this teaching method [16]. In the FMUP, Portugal, the curricular plan of the Integrated Master’s Degree in Medicine was restructured in 2013 and again in 2021. In the curricular plan before 2013, Histology and Embryology were taught together but in two separate curricular units, named “Basic Histology and Embryology” and “Histology and Embryology of Organs and Systems” that ran in the second year of the 6 years course. In the curricular plan from 2013 to 2021, Histology and Embryology were integrated with other areas of fundamental knowledge, namely Anatomy and Physiology in the first and second years of the course. In the recent curricular definition in our medical school, Histology was proposed to be taught separately from Embryology to allow students a better comprehension of both. Throughout the world, innovations for teaching Histology and Embryology are emerging, by using of animations to teach Histology as part of an interactive multimedia program called “HistoQuest” [17], digital slides and digital microscopes [18, 19] and gamification [20]. Some of these methods were intensively used during Covid-19 pandemics [21], along with online synchronous and asynchronous lectures, implementation of learner-centered curriculum (focused on active learning and in create an environment that balanced student independence with teacher guidance) and along with the use of virtual microscopes [21].

Medical students frequently struggle to recognise the relevance of Histology and Embryology for their future practice as medical doctors and can be unmotivated to study these subjects. To increase motivation, students should be co-agents in the construction of their own medical knowledge and their opinions should be considered [22]. This allows to increase the intrinsic motivation of medical students by expanding their autonomy and competences. The combination of good intrinsic motivation, which provides highly productive and spontaneous learning, with good extrinsic motivation, based on an integrated regulation (with values of learning fully assimilated into self) was proposed to increase the motivation of medical students [23]. Additionally, the MeSAGE (Medical Student Alliance for Global Education), a global collaboration of student organizations working together to lead the next wave of the medical education revolution, proposed that students should play an active and responsible role in the process of improving medical curriculum and defining teaching/learning methodologies [24]. This was also demonstrated in our medical school by Ramalho et al. [25], who highlighted the role of medical students in the electives of the medical curriculum.

Recently, two studies evaluated the attitudes of medical students at European universities towards the clinical importance of Histology [26] and of Embryology [27]. Their results show that medical students fail to recognise the clinical importance of those subjects, except for the Portuguese medical school enrolled in the survey (Faculty of Medical Sciences, NOVA University of Lisbon). There are still few studies that focus on medical students’ perceptions about the role of Histology and Embryology in the medical curriculum and the most suitable teaching methods. Taking this into account we performed a large survey that collected the perceptions of a representative population of FMUP’s students regarding the role of Histology and Embryology in the medical curriculum, namely the teaching methods, clinical relevance and integration with other subjects of the medical course.

## Material and methods

### Study design and participants

This study was submitted and approved by the Ethics and Research Committee of the São João Hospital/FMUP (protocol number CE 205–21). This study is a large survey that was conducted during 1st of June and 16th July of 2021, during the preparation to adjust the medical curriculum in FMUP.

Informed consent was obtained from each participant, after explanation of the study’s objectives. All participants were assured of the confidentiality of the information to be collected and that they were free to decline to participate in the study. Data acquisition followed the self-administered questionnaire without the intervention of the specific person and it did not contain any identifying data of the participants to ensure confidentiality. Only filled questionnaires were included for the data analysis. Inclusion criteria was the attendance of medical students in the third, fourth, fifth or sixth years of the medical course, aged 18 years or older, and understanding and agreement to anonymously participate in the study.

### Data collection instrument

Medical students at FMUP were invited to complete a structured and anonymous online questionnaire (using Google Forms), consisting of three parts. The first part comprised socio-demographic characteristics of the participants, including age, sex and year of the course. Only students of the third, fourth, fifth and sixth years of the course were contacted because they already studied Histology and Embryology in the first and second years of the course. The second part, comprising 24 questions, evaluated the participant’s perceptions about the role of Histology and Embryology in the medical curriculum and clinical learning; participants answered the questions using a 5-point Likert scale (1 being “strongly disagree” and 5 being “strongly agree”). The 24 questions were divided in 3 groups (one comprising questions related with Histology, another comprising questions related with Embryology and a final group with questions related with teaching methods). Some of the questions were already used in previous studies which included another Portuguese medical school [26, 27]. The last part was a short-answer open question asking participants to indicate some suggestions and opinions regarding the teaching of Histology and Embryology and the role of these subjects in the medical curriculum, namely in clinical learning. The answers to the questionnaire were given sequentially, so that only the open question could be left unanswered. As referred above, several questions used in the questionnaire were already used in another Portuguese medical school, namely questions 1, 2, 3 and 4 [26] and questions 11, 12, 13 and 14 [27]. Nevertheless, to further ensure the adequation to our students, the overall questionnaire was pretested on 15 randomly selected students of FMUP from the third to sixth year of the course. These students did not participate in the final survey. A Cronbach’s alpha analysis was performed and an alpha value of 0.79 was obtained, which means that the questionnaire has an acceptable internal consistency [28]. The pretested questionnaire was equal to the final questionnaire.

### Data analysis

The study included a descriptive and a comparative analysis. The statistical analysis were performed using the Microsoft Excel version 16.52 (for descriptive analysis and for creating graphs) and using Statistical Package for Social Sciences (SPSS) version 28.0 (for comparative analysis). The Chi-square test was performed using raw data to make comparisons among categorical variables. The open question was qualitatively analysed and summarised by two researchers with Kappa test used to verify concordance. The *p* value was set at 0.05.

## Results

A total of four hundred and sixty-two (*n* = 462) answers to the questionnaire were obtained, which is a large and representative sample of students, with almost 50% adherence by the eligible population (980 students). The socio-demographic characteristics of the sample are shown in Table 1.

**Table 1:** Socio-demographic characteristics of the sample

Characteristics	Total sample	Third year sample	Fourth year sample	Fifth year sample	Sixth year sample
N	462	126	136	103	97
Age (years)*	22.6 [22.4; 22.8]	21.5 [21.0; 22.0]	22.2 [21.7; 22.7]	23.3 [22.9; 23.7]	24.1 [23.7; 24.5]
Sex, n (%)					
Female	327 (70.8%)	94 (74.6%)	92 (67.7%)	80 (77.7%)	61 (62.9%)
Male	134 (29.0%)	32 (25.4%)	43 (31.6%)	23 (22.3%)	36 (37.1%)
Other	1 (0.2%)	0 (0.0%)	1 (0.7%)	0 (0.0%)	0 (0.0%)

*Arithmetic mean (95% CI). *N *Number

**Fig. 1:**
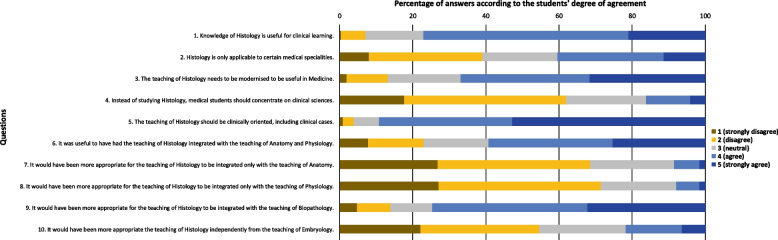
Total sample answers to the questions related with Histology

**Fig. 2:**
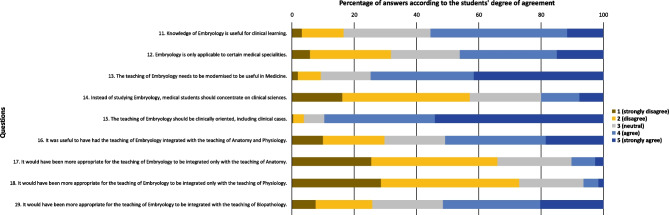
Total sample answers to the questions related with Embryology

**Fig. 3:**
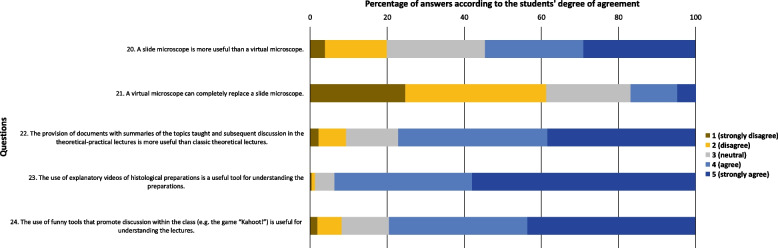
Total sample answers to the questions related with teaching methodologies

A descriptive analysis of the answers to the 24 questions in the second part of the questionnaire was performed for the total sample and for each of the sub samples (third year sample, fourth year sample, fifth year sample and sixth year sample). The results of the total sample descriptive analysis are summarised in Figs. 1, 2 and 3.

Regarding the questions related with Histology (Fig. 1), most students agreed (answer “strongly agree” or “agree”) with questions “Knowledge of Histology is useful for clinical learning” (77.1%), “The teaching of Histology needs to be modernised to be useful in Medicine” (66.9%), “The teaching of Histology should be clinically oriented, including clinical cases” (89.2%), “It was useful to have had the teaching of Histology integrated with the teaching of Anatomy and Physiology” (59.3%) and “It would have been more appropriate for the teaching of Histology to be integrated with the teaching of Biopathology” (74.7%). Most students disagreed (answer “strongly disagree” or “disagree”) with questions “Instead of studying Histology, medical students should concentrate on clinical sciences” (61.9%), “It would have been more appropriate for the teaching of Histology to be integrated only with the teaching of Anatomy” (68.6%), “It would have been more appropriate for the teaching of Histology to be integrated only with the teaching of Physiology” (71.5%) and “It would have been more appropriate the teaching of Histology independently from the teaching of Embryology” (54.6%). The answers to question “Histology is only applicable to certain medical specialities” were more balanced between agreement and disagreement than the remaining ones.

Regarding the questions related with Embryology (Fig. 2), most students agreed with questions “Knowledge of Embryology is useful for clinical learning” (55.4%), “The teaching of Embryology needs to be modernised to be useful in Medicine” (74.7%), “The teaching of Embryology should be clinically oriented, including clinical cases” (89.6%), “It was useful to have had the teaching of Embryology integrated with the teaching of Anatomy and Physiology” (50.9%) and “It would have been more appropriate for the teaching of Embryology to be integrated with the teaching of Biopathology” (51.5%). Most students disagreed with questions “Instead of studying Embryology, medical students should concentrate on clinical sciences” (57.1%), “It would have been more appropriate for the teaching of Embryology to be integrated only with the teaching of Anatomy” (66.0%) and “It would have been more appropriate for the teaching of Embryology to be integrated only with the teaching of Physiology” (73.0%). The answers to the question “Embryology is only applicable to certain medical specialities” were more balanced.

Regarding the questions related with teaching methodologies (Fig. 3), most students agreed with questions “A slide microscope is more useful than a virtual microscope” (54.5%), “The provision of documents with summaries of the topics taught and subsequent discussion in the theoretical-practical lectures is more useful than classical theoretical lectures” (77.0%), “The use of explanatory videos of histological preparations is a useful tool for understanding the preparations” (93.7%) and “The use of funny tools that promote the discussion within the class (e.g. the game “Kahoot!”) is useful for understanding the lectures” (79.4%). Most students disagreed with question “A virtual microscope can completely replace a slide microscope” (61.3%).

To summarise the initial descriptive analysis, most students agreed that Histology is clinically relevant and that it should be taught with a clinical perspective and that Histology should be integrated with Pathology. The students also felted strongly that teaching of Embryology should be modernised and clinically oriented. The students felted strongly that a virtual microscope could not replace a slide microscope.

Additionally, an arithmetic mean of the answers to each question was performed for each sub sample answers. The results are in line with what was obtained for the total sample; detailed results are shown in Table 2, Table 3 and Table 4.

**Table 2:** Sub samples average (arithmetic mean with 95% confidence interval) of answers to the questions related with Histology

Questions	Answers			
	Third year sample	Fourth year sample	Fifth year sample	Sixth year sample
1. Knowledge of Histology is useful for clinical learning.	3.9 [3.8; 4.0]	3.9 [3.8; 4.0]	3.9 [3.7; 4.1]	3.9 [3.7; 4.1]
2. Histology is only applicable to certain medical specialities.	3.3 [3.1; 3.5]	2.8 [2.6; 3.0]	3.0 [2.8; 3.2]	3.0 [2.8; 3.2]
3. The teaching of Histology needs to be modernised to be useful in Medicine.	3.9 [3.7; 4.1]	4.0 [3.8; 4.2]	3.8 [3.6; 4.0]	3.5 [3.3; 3.7]
4. Instead of studying Histology, medical students should concentrate on clinical sciences.	2.5 [2.3; 2.7]	2.3 [2.1; 2.5]	2.5 [2.3; 2.7]	2.4 [2.2; 2.6]
5. The teaching of Histology should be clinically oriented, including clinical cases.	4.2 [4.0; 4.4]	4.5 [4.4; 4.6]	4.4 [4.3; 4.5]	4.4 [4.2; 4.6]
6. It was useful to have had the teaching of Histology integrated with the teaching of Anatomy and Physiology.	3.7 [3.5; 3.9]	3.6 [3.4; 3.8]	3.5 [3.3; 3.7]	3.3 [3.0; 3.6]
7. It would have been more appropriate for the teaching of Histology to be integrated only with the teaching of Anatomy.	2.3 [2.1; 2.5]	2.0 [1.8; 2.2]	2.2 [2.0; 2.4]	2.1 [1.9; 2.3]
8. It would have been more appropriate for the teaching of Histology to be integrated only with the teaching of Physiology.	2.2 [2.0; 2.4]	2.1 [1.9; 2.3]	2.1 [1.9; 2.3]	2.0 [1.8; 2.2]
9. It would have been more appropriate for the teaching of Histology to be integrated with the teaching of Biopathology.	4.0 [3.8; 4.2]	3.8 [3.6; 4.0]	3.7 [3.5; 3.9]	3.9 [3.7; 4.1]
10. It would have been more appropriate the teaching of Histology independently from the teaching of Embryology.	2.5 [2.3; 2.7]	2.6 [2.4; 2.8]	2.6 [2.4; 2.8]	2.4 [2.2; 2.6]

**Table 3:** Sub samples average (arithmetic mean with 95% confidence interval) of answers to the questions related with Embryology

Questions	Answers			
	Third year sample	Fourth year sample	Fifth year sample	Sixth year sample
11. Knowledge of Embryology is useful for clinical learning.	3.3 [3.1; 3.5]	3.6 [3.4; 3.8]	3.6 [3.4; 3.8]	3.4 [3.2; 3.6]
12. Embryology is only applicable to certain medical specialities.	3.6 [3.4; 3.8]	2.9 [2.7; 3.1]	3.0 [2.8; 3.2]	3.4 [3.2; 2.6]
13. The teaching of Embryology needs to be modernised to be useful in Medicine.	4.1 [3.9; 4.3]	4.2 [4.0; 4.4]	4.0 [3.8; 4.2]	3.8 [3.6; 4.0]
14. Instead of studying Embryology, medical students should concentrate on clinical sciences.	2.7 [2.5; 2.9]	2.4 [2.2; 2.6]	2.5 [2.3; 2.7]	2.6 [2.4; 2.8]
15. The teaching of Embryology should be clinically oriented, including clinical cases.	4.2 [4.1; 4.3]	4.5 [4.4; 4.6]	4.4 [4.3; 4.5]	4.4 [4.2; 4.6]
16. It was useful to have had the teaching of Embryology integrated with the teaching of Anatomy and Physiology.	3.3 [3.1; 3.5]	3.4 [3.2; 3.6]	3.4 [3.2; 3.6]	3.1 [2.8; 3.4]
17. It would have been more appropriate for the teaching of Embryology to be integrated only with the teaching of Anatomy.	2.3 [2.1; 2.5]	2.1 [1.9; 2.3]	2.3 [2.1; 2.5]	2.2 [2.0; 2.4]
18. It would have been more appropriate for the teaching of Embryology to be integrated only with the teaching of Physiology.	2.2 [2.0; 2.4]	2.1 [1.9; 2.3]	2.0 [1.8; 2.2]	2.0 [1.8; 2.2]
19. It would have been more appropriate for the teaching of Embryology to be integrated with the teaching of Biopathology.	3.3 [3.1; 3.5]	3.4 [3.2; 3.6]	3.3 [3.1; 3.5]	3.5 [3.3; 3.7]

**Table 4:** Sub samples average (arithmetic mean with 95% confidence interval) of answers to the questions related with teaching methodologies

Questions	Answers			
	Third year sample	Fourth year sample	Fifth year sample	Sixth year sample
20. A slide microscope is more useful than a virtual microscope.	3.4 [3.2; 3.6]	3.8 [3.6; 4.0]	3.5 [3.3; 3.7]	3.6 [3.4; 3.8]
21. A virtual microscope can completely replace a slide microscope.	2.5 [2.3; 2.7]	2.4 [2.2; 2.6]	2.3 [2.1; 2.5]	2.3 [2.1; 2.5]
22. The provision of documents with summaries of the topics taught and subsequent discussion in the theoretical-practical lectures is more useful than classic theoretical lectures.	4.1 [3.9; 4.3]	4.0 [3.8; 4.2]	4.1 [3.9; 4.3]	3.9 [3.7; 4.1]
23. The use of explanatory videos of histological preparations is a useful tool for understanding the preparations.	4.6 [4.5; 4.7]	4.5 [4.4; 4.6]	4.4 [4.3; 4.5]	4.4 [4.3; 4.5]
24. The use of funny tools that promote discussion within the class (e.g. the game “Kahoot!”) is useful for understanding the lectures.	4.2 [4.0; 4.4]	4.2 [4.0; 4.4]	4.2 [4.0; 4.4]	3.9 [3.7; 4.1]

A comparative analysis was then performed. For this analysis, 6 questions were selected from the second part of the questionnaire (3 questions related with Histology and 3 questions related with Embryology). In this analysis the answers “strongly disagree” and “disagree” were aggregate in “disagree” and the answers “agree” and “strongly agree” in “agree” (the answer “neutral” was maintained) and the responses of the basic year (third year in which the clinical contact is still incipient) with the clinical years (fourth to sixth were the students are mostly in the hospital) were compared. The results are shown in Table 5. Statistically significant differences were obtained for questions 2 (*p* = 0.016), 5 (*p* = 0.023) and 12 (*p* < 0.001), showing differences in perspectives of students of the basic year and clinical years.

**Table 5:** Comparative analysis between basic year (third) and clinical years (fourth, fifth and sixth)

Selected questions	Answer	Basic year (%)	Clinical years (%)	*p* value*
1. Knowledge of Histology is useful for clinical learning.	Disagree	6.3	7.4	0.790
	Neutral	17.5	15.2	
	Agree	76.2	77.4	
2. Histology is only applicable to certain medical specialities.	Disagree	28.6	42.9	0.016^§^
	Neutral	22.2	19.9	
	Agree	49.2	37.2	
5. The teaching of Histology should be clinical oriented, including clinical cases.	Disagree	7.9	2.4	0.023^§^
	Neutral	6.3	7.1	
	Agree	85.7	90.5	
11. Knowledge of Embryology is useful for clinical learning.	Disagree	20.6	15.2	0.154
	Neutral	31.0	26.8	
	Agree	48.4	58.0	
12. Embryology is only applicable to certain medical specialities.	Disagree	17.5	37.2	< 0.001^§^
	Neutral	24.6	21.1	
	Agree	57.9	41.7	
15. The teaching of Embryology should be clinical oriented, including clinical cases.	Disagree	6.3	3.0	0.249
	Neutral	6.3	6.5	
	Agree	87.3	90.5	

*Chi-square test. ^§^Statistically significative differences

Seventy-eight students (*n* = 78) answered to the short-answer open question. All answers were analysed independently by two of the authors; a concordance index of 81.6% were obtained. Regarding Histology, students highlighted its clinical importance and its role as the basis to understand Biopathology (e.g. “Histology is useful in the training of future doctors, allowing a better understanding of Biopathology”). Probably because of that, most of students recommended an integration of Histology with Biopathology (e.g. “I think Histology and Biopathology should be taught together”) and recommended the inclusion of clinical cases in Histology teaching (e.g. “Using clinical cases would be very advantageous for the study of Histology”). Concerning Embryology, students also highlighted its clinical importance (e.g. “Embryology is useful in medical training because it helps to understand human development”) and recommended the inclusion of clinical cases in Embryology teaching (e.g. “The teaching of Embryology should favour integration with clinical cases”). However, in this case, they had not recommended an integration of Embryology with Biopathology, instead of that, students have the opinion that Embryology should be teach alone, because of her complexity (e.g. “Embryology is a very specific area and should therefore be taught as a single subject”). Students also said that Embryology teaching should be modernised (e.g. “In the teaching of Embryology it would be very useful to make use of more modern teaching methods that include, for instance, three-dimensional videos”). Regarding teaching methodologies, students said that they prefer the teaching of Histology and Embryology independently from other basic sciences teaching, such as Anatomy or Physiology (e.g. “I found that when integrated with Anatomy or Physiology, Histology and Embryology were undervalued and therefore should be taught independently”) and most of the students highlighted the importance of the practical component, including the microscopic observation (e.g. “The practical component using slide microscopes is fundamental to understand Histology and Embryology”), and the benefits of using new technologies in Histology and Embryology teaching (e.g. “The most important thing is to modernise the way lessons are given, with the incorporation of new technologies”).

## Discussion

In the present study, we collected new data on the perceptions of a representative sample of students from a Portuguese medical school (the Faculty of Medicine of the University of Porto) regarding the role of Histology and Embryology in the medical curriculum. The present large survey, by means of a structured and anonymous online questionnaire, demonstrated that most students recognise the clinical importance of Histology and Embryology, both in the structured questionnaire and in the short-answer open question. These results were even more expressive than the previous European studies [26, 27]. Curiously, in those studies, students from another Portuguese university (Nova Medical School in Lisbon) also strongly reported the clinical importance of Histology and Embryology, in a manner more obvious than the other European medical schools. These results indicate that historical and cultural backgrounds should be considered in medical education.

It was also highlight by the students that Histology and Embryology teaching should be clinically oriented, including clinical cases, which agrees with the conclusions and suggestions of Zaletel et al. [29]. Furthermore, it is known that the educational process may be modernised, and that Histology and Embryology teaching are not exception, as demonstrated and emphasised in the short-answer open question by our students and also highlighted by Gribbin et al. [13]. Within the curriculum reform that occurred in 2021 in our medical school there was a call for teaching medical student content that is relevant to their future clinical work [30] and to include the use of clinical cases may promote the ability to understand complex clinical problems faced by the students later in the medical course [31].

In relation to the questions 2 (“Histology is only applicable to certain medical specialities”), 5 (“Teaching of Histology should be clinically oriented, including clinical cases”) and 12 (“Embryology is only applicable to certain medical specialities”) we found that basic year medical students’ perceptions and clinical years medical students’ perceptions are significantly different. In fact, clinical years students are more likely to disagree that Histology and Embryology are only applicable to certain medical specialities and are more likely to agree that teaching of Histology should be clinical oriented, including clinical cases, than basic year medical students. That is, students with a better knowledge of the clinical reality recognise a greater transversality of Histology and Embryology in the clinical context, which is probably due to the higher contact with clinical work of those students. These results were different from the study of Zaletel et al. [29] which might be due to differences in the structure of the two medical courses. It may be important to present the Histology and Embryology courses with real clinical examples that may motivate the younger students to the clinical importance of those subjects since the beginning of the course. The enrolment of students from more advanced years in teaching in the first years of the medical course may also help to increase the clinical perspectives of their younger colleagues, because the older students frequently make a bridge between clinical sciences and basic contents. In fact, it has been proposed that enrolling older students (that are more advanced in the medical courses), as teachers, based on a Peer Assisted Learning [32], may enable to bridge the gap between being a university student and being a clinician [33] and that students feel more at ease with an older colleague as teacher than with senior professors [14]. This fact, together with the engagement of students as co-agents in the construction of their own medical knowledge, allowing them to express their opinions [22], is fundamental to increase the student’s motivation in basic medical education. However, in the current study the students that replied to the questionnaire already finished Histology and Embryology herein the consequences in direct motivation of future students attending Histology and Embryology would not be intrinsic and might be derived from the participation of older colleagues as professors in the classes.

We found that medical students at FMUP prefer the teaching of Histology and Embryology independently from other basic sciences teaching, specifically Anatomy and Physiology. A large majority of students agree that the integration between Histology and Biopathology could be positive. Previous studies showed that the simple implementation of a few histopathology examples can yield a tremendous improvement in basic years medical students’ understanding, enjoyment, and engagement in practical Histology classes [34]. Histology seems to be easily related to Biopathology, an aspect that could determine the future of the teaching in our, and maybe other, medical school. This is not the case of Embryology, were the integration with Biopathology was not recognised as positive. This shows that Embryology per se is a difficult and complex subject that should be learnt at its own pace. Integrating Embryology with other basic medical sciences is not appreciated by the students, which favours teaching it independently. Most of these opinions of medical students were incorporated in the review of the medical curriculum in our school.

Another important aspect to be discussed among medical educators is the appropriate usage of new technologies in the teaching process. It is well recognised that the use of computers and mobile phones in gamification activities generates more interest and participation in the classes [17, 20, 21]. However, virtual slides and virtual microscopes [18, 19] seems to be a controversial topic under discussion. Some authors claimed that the transition from slide to virtual microscopes is positive in their teaching context [18] and a meta-analysis study revealed that the pedagogical approach of virtual microscopes is modestly superior to that of slide microscopes and is preferred by learners [35]. Our study demonstrated a preference for slide microscopes. We believe that use a slide microscope is an excellent opportunity for improving students’ skills. This is related to the process of using a microscope per se and the perception of different slides of a tissue, each of the slides with different peculiarities, including sharing these variations among students, increasing learning by pairs. Meanwhile, the digital microscope usually has only one slide per tissue, being useful for home study or even for teaching in pandemic scenarios. Additionally, the current excessive screen time of medical students namely in the pandemic phase may influence in the slide microscope preference. In fact, slide microscopes have become the “novel feature” in the Histology and Embryology classroom, because screens are currently so ubiquitous.

There are some limitations to our study. First, despite a very representative sample we were not able to assess the perceptions of all eligible students. Second, most of students at FMUP only had contact with the Histology and Embryology teaching method taught at FMUP, which may bias the answers. Regarding the use of the Likert scale, which is a widely used scaling technique namely in medical education research [36], we are aware of some of its limitations, which were pointed out [36–38] and may include the difficulty to interpret neutral opinions and the possibility of different interpretations of the same item by different people in the sample. The later was probably minimised because our sample is not diverse in what concerns cognitive abilities and literacy. The main reason to use the Likert scale is the high adherence of our medical students to questionnaires using that scaling technique [39].

This study appraises the perceptions of medical students of our faculty about the role of Histology and Embryology in the medical curriculum and about the teaching methods being used in Histology and Embryology. However, it is not possible to generalise the results to other institutions. The results of the present study, nonetheless, helped in some modifications in the Histology and Embryology of FMUP, namely the introduction of more clinically oriented discussions and the combination of slide and digital microscopes. Further studies in this area are necessary. First it is important that all institutions keep performing audits to the students to allow an inclusive and current teaching program in all subjects and second it is important that this can contribute to constantly improve the methodology teaching used in Histology and Embryology, specifically in the medical teaching. The effects of adjusting the curricular contents between Histology and Biopathology in our Faculty should be considered in a near future. We also want to evaluate if the changes in teaching Embryology, namely increasing the clinical implications of this subject, better prepares the students for the clinical part of the course, namely Obstetrics and other medical specialities.

## Conclusions

The present study contributes to the debate about the evolution of medical curriculum and about the new challenges in this topic; it also allows us to know the medical students’ perceptions, which are useful for improving the medical curriculum. Consulting and involving medical students in the development of the medical curriculum can be positive for all the agents involved in the teaching process, both students and their professors.

## Supplementary Information

**Additional file 1.**

## Data Availability

Anonymised raw data used in the statistical analysis can be found in the supplementary material.

## Abbreviations

FMUP: (Faculty of Medicine of the University of Porto)
MeSAGE: (Medical Student Alliance for Global Education)
